# Investigation of Atomic‐Scale Mechanical Behavior by Bias‐Induced Degradation in Janus and Alloy Polymorphic Monolayer TMDs via In Situ TEM

**DOI:** 10.1002/smsc.202300129

**Published:** 2023-11-21

**Authors:** Hsin-Ya Sung, Chieh-Ting Chen, Yi-Tang Tseng, Yu-Lun Chueh, Wen-Wei Wu

**Affiliations:** ^1^ Department of Materials Science and Engineering National Yang Ming Chiao Tung University Hsinchu 30010 Taiwan; ^2^ Department of Materials Science and Engineering National Tsing Hua University Hsinchu 30010 Taiwan; ^3^ Colleage of Semiconductor Research National Tsing-Hua University Hsinchu 30013 Taiwan; ^4^ Department of Physics National Sun Yat-Sen University Kaohsiung 80424 Taiwan; ^5^ Advanced Semiconductor Technology Research Center Hsinchu 30078 Taiwan

**Keywords:** alloyed transition-metal dichalcogenides (TMDs), high-resolution transmission electron microscopes/scanning transmission electron microscopes (TEMs/STEMs), in situ biasings, Janus TMDs, Joule heatings, mechanical properties

## Abstract

The 2D Janus transition‐metal dichalcogenides (TMDs) and alloyed TMDs are a widely studied emerging class of 2D materials that have been extensively used in electronic devices because of their excellent electronic, optical, and mechanical properties. The properties and behaviors of 2D‐materials‐based devices, such as the electrical breakdown caused by structural failure, are significant issues that have drawn considerable attention. In this study, the electrical behavior of polymorphic molybdenum sulfide selenide (MoSSe) devices is studied *via* in situ biasing experiments and recorded using transmission electron microscopy (TEM) at the atomic scale. The selenization temperature is a key factor in the phase transition of the material, which further affects the electrical and mechanical properties of MoSSe. The effects of electron‐beam irradiation and bias voltage are also discussed through a combination of experiments and theory. Quantifying the defect coverage and defect size also helps us to understand the behavior of material degradation. Furthermore, Cs‐corrected scanning TEM is utilized to identify the evolution of the morphology. The fracture morphology of the synthesized structure also varies with the application of high voltage. The cracks and defects caused by Joule heating are studied in terms of fracture type and size.

## Graphical abstract

Janus and alloy polymorphic monolayer molybdenum sulfide selenide (MoSSe) prepared at different selenization temperatures exhibits different electrical and mechanical properties after biasing. In the structural degradation of MoSSe, powerful in situ transmission electron microscope (TEM) and annular dark‐field scanning TEM are used to understand the individual effects of electron beams and bias.

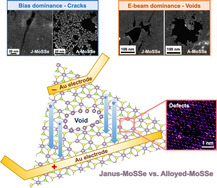

## 1 Introduction

Since the discovery of graphene in 2004,^[^ ^1^ ^]^ 2D materials, such as hexagonal boron nitride (h‐BN), transition‐metal dichalcogenides (TMDs), transition‐metal oxides, and black phosphorus, have attracted increasing attention. Among these 2D materials, TMDs have attracted considerable attention, with MoS_2_ and WS_2_ as representative materials. Their tunable bandgap and outstanding optical and electrical properties^[^ ^2^, ^3^, ^4^ ^]^ result in their potential applications in novel electronic and optoelectronic devices, such as gas sensors,^[^ ^5^, ^6^ ^]^ memory devices,^[^ ^7^, ^8^ ^]^ field‐effect transistors,^[^ ^9^, ^10^, ^11^ ^]^ and photodetectors,^[^ ^12^, ^13^ ^]^ over the last decade. Controlling the number of layers in 2D materials can change the energy band variation from the indirect bandgap of a multilayer to the direct bandgap of a single layer, further affecting the electrical performance of the device.

Graphene exhibits characteristics of a semimetal, whereas h‐BN functions as a wide gap semiconductor. The h‐BN has a layered structure similar to graphene with an almost same lattice constant, the potential of developing a monolayer boron carbonitride (BCN) system has been envisaged, wherein the gap could be adjusted by varying the concentration of each component. Both theory^[^ ^14^ ^]^ and experiments^[^ ^15^ ^]^ have demonstrated that 2D BCN materials face challenges in terms of thermodynamic stability, with h‐BN and graphene, tend to segregate.^[^ ^16^ ^]^ On the contrary, the TMD families (MX_2_: M = Mo, W, etc.; X = S, Se, Te) makes them great candidates to form van der Waals (vdW) alloys that will not suffer from phase separation.^[^ ^17^ ^]^ Moreover, vdW heterostructure can also be effectively used in phase‐change heterostructure, which the electrical‐pulse‐driven 2D order–disorder phase transitions, and it affects its electrical performance.^[^ ^18^ ^]^

The unique band structure of monolayered MoS_2_, with its breakdown of inversion symmetry, provides outstanding opportunities for applications in optical and electronic devices. Theory suggests that the possibility of adjusting the band structure of MoS_2_ by replacing sulfur atoms with alternative chalcogen elements, thus forming TMD alloys. Moreover, the mixing energy of the alloy is low, and it is a thermodynamically stable phase with a direct energy gap. This enables a tunable mechanism to change the physical and chemical properties of MoS_2_.^[^ ^19^ ^]^ Further, 2D Janus TMDs (J‐TMDs) are sandwiched between two different chalcogen layers, resulting in broken mirror symmetry along the out‐of‐plane direction.^[^ ^17^ ^]^ The asymmetry of J‐TMDs also provides potential for application in electrical devices owing to vdW interlayer coupling and charge transfer in vdW heterostructures.^[^ ^20^, ^21^, ^22^ ^]^ Furthermore, in 2D alloyed TMDs (A‐TMDs), chalcogen atoms were randomly distributed in the structure. Theoretical calculations indicated that ordered phases have lower energies than random phases. Random structures usually have larger entropies owing to the temperature conditions of the synthesis process.^[^ ^23^ ^]^

However, methods for the synthesis of 2D J‐TMDs and A‐TMDs have not yet been widely established. In previous studies, Janus molybdenum sulfide selenide (J‐MoSSe) was successfully grown by controlling the substitution of S or Se atoms on the surface of a MoS_2_ or MoSe_2_ layer with Se or S atoms, respectively.^[^ ^19^, ^24^, ^25^ ^]^ In addition, the alloyed WSSe monolayers were synthesized using a colloidal growth protocol.^[^ ^26^ ^]^ In this study, we explored the structural difference between 2D J‐TMDs and 2D A‐TMDs, which can directly affect their electrical performances.

Currently, owing to the increasing demand for electronic devices, investigating the performance of materials in electronic and optoelectronic devices to evaluate the technical feasibility of 2D‐based devices for practical applications is essential. Electrical breakdown associated with material damage is a critical issue in device failure. However, few studies have mentioned that the relationship between the different phase structures and the mechanical properties of the materials will further affect the electrical performance of the device. According to previous studies, voids and cracks are formed by biasing.^[^ ^27^, ^28^ ^]^ In addition, the fracture of materials is caused by the disconnection of atomic bonds. Brittle fracture is associated with the rapid clean cleavage of bonds, and ductile fracture generally involves plastic deformation around the tip front, which slows down crack propagation.^[^ ^29^ ^]^ Additionally, defects in materials also further affect the mechanical properties of devices. Low‐density vacancy defects cause crack deflection, while high‐density defects alter the fracture mechanism from brittle to ductile, which result in the enhanced fracture toughness of MoS_2_ even exceeding that of graphene.^[^ ^30^ ^]^ The breakdown behavior of 2D‐based devices has been widely studied through current–voltage characteristic curves,^[^ ^31^, ^32^ ^]^ and some studies have used a simulation method to detect the temperature changes of the devices caused by the Joule heating effect.^[^ ^33^ ^]^ Previous studies have mainly discussed the effect of stress on material fracture,^[^ ^34^, ^35^ ^]^ but direct observations of dynamic changes of tip growth combined with bias effects in materials are rare.

In situ transmission electron microscopy (TEM) is a powerful technique for directly observing the response of the sample to external stimuli such as heat and bias^[^ ^36^, ^37^, ^38^, ^39^, ^40^, ^41^, ^42^ ^]^ and provides high‐resolution images for the atomic‐scale understanding of the microstructure. Over the past decade, in situ TEM/scanning TEM (STEM) experiments have been used to realize an in‐depth understanding of electron transport in resistive random‐access memory nanodevices,^[^ ^43^, ^44^, ^45^ ^]^ nanotwinned copper,^[^ ^46^ ^]^ solid‐state diffusion of metal oxides,^[^ ^47^, ^48^ ^]^ nanostructure evolution in liquids,^[^ ^49^ ^]^ and lithium‐ion reactions that occur in batteries.^[^ ^50^ ^]^ However, relevant research on the in situ biasing of 2D materials is scarce in the literature.^[^ ^51^, ^52^, ^53^ ^]^ Therefore, we focused on *in situ* observations via high‐resolution TEM (HRTEM) to examine the structural evolution of the two phases of MoSSe devices under biased conditions. After the *in situ* biasing process, large‐area analysis and observations were performed using annular dark‐field STEM (ADF*‐*STEM) to provide a complete picture of the evolution. In this case, the two main factors, electron‐beam irradiation and biasing, are discussed based on the experimental results and theoretical analysis. Samples prepared at different selenization temperatures formed different phases, and the variations in the mechanical properties and electrical behaviors after biased were analyzed. Notably, we utilized atomic‐resolution STEM images to identify crack propagation. The different phases of MoSSe were investigated using a combination of experiments and theory to better understand the relationship between defect concentration and bias. Finally, detailed atomic structure and elemental analyses were performed to obtain direct evidence of the behavioral mechanism.

## 2 Results and Discussion

**Figure 1:**
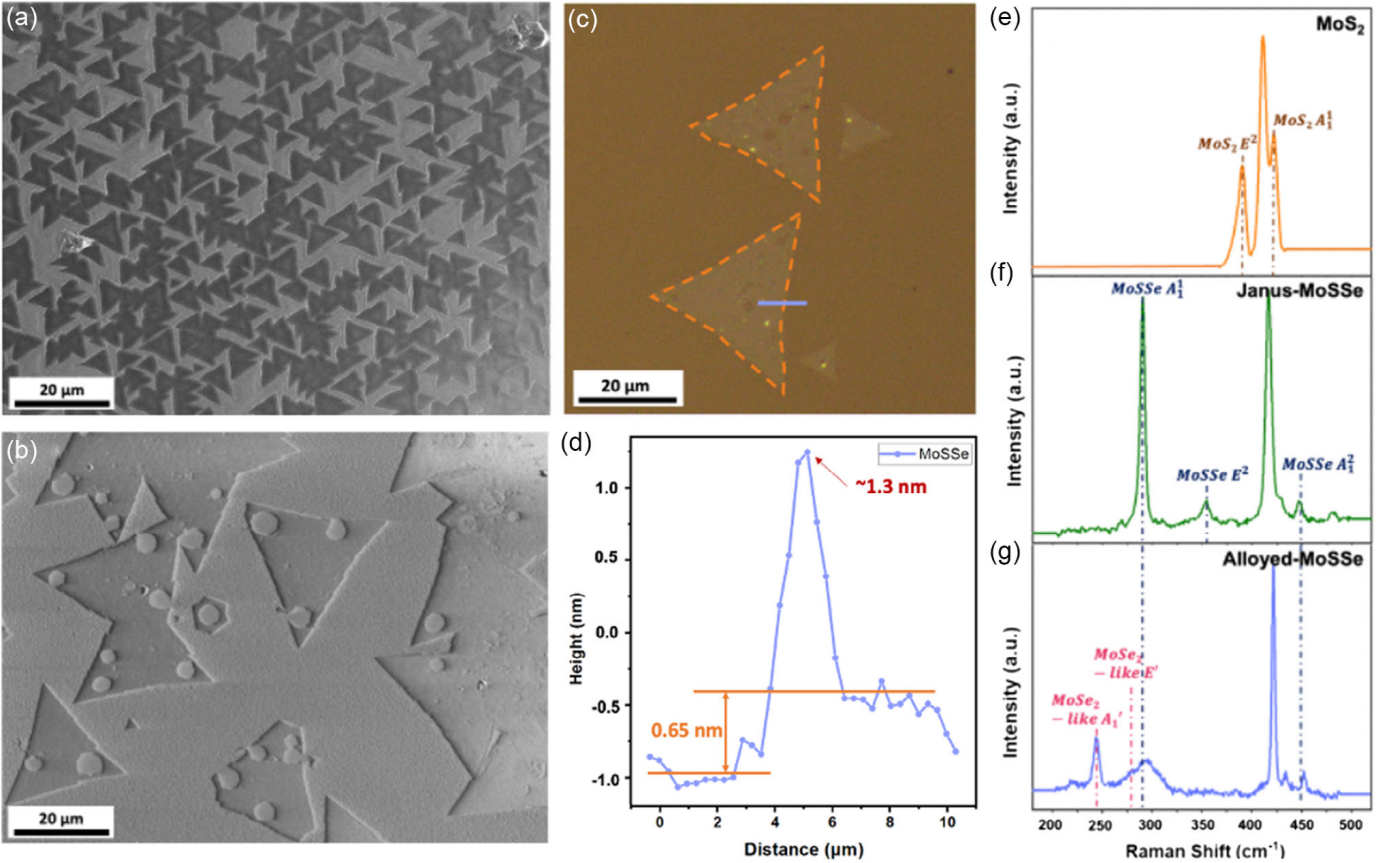
Characterization of as‐grown MoS_2_ and molybdenum sulfide selenide (MoSSe). a) Scanning electron microscopy (SEM) image of as‐grown MoS_2_. b) SEM image of as‐grown Janus‐MoSSe (J‐MoSSe) sample. c) Optical microscope (OM) image of the as‐grown J‐MoSSe sample. d) Atomic force microscopy height profile of J‐MoSSe (along with the purple line in (c)), giving an average thickness of ≈0.65 nm. e) Raman spectrum of as‐grown MoS_2_. f) Raman spectrum of as‐grown J‐MoSSe. g) Raman spectrum of as‐grown alloyed‐MoSSe (A‐MoSSe). The substrate signal of sapphire (Al_2_O_3_) is approximately 420 cm^−1^.

High‐quality monolayer MoS_2_ was grown on sapphire substrates by chemical vapor deposition (CVD) process in a horizontal furnace tube with two heating zones (Figure S1a, Supporting Information). Crystalline‐atomic‐layer MoSSe was obtained through a plasma‐assisted chemical vapor reaction (PACVR) process in a vertical furnace tube with three different chambers to replace the S atoms in MoS_2_ with Se atoms (Figure S1b, Supporting Information). J‐MoSSe and alloyed MoSSe (A‐MoSSe) could be synthesized by controlling the temperature of the Se tank heated to 300 and 450 °C, respectively. A detailed characterization of the as‐grown MoS_2_ and J‐MoSSe samples is shown in **Figure** **1**a,b, and S2, Supporting Information, shows the scanning electron microscopy (SEM) image of the high density and high quality of MoS_2_ and MoSSe. Figure 1c,d shows the optical microscope (OM) image and atomic force microscopy (AFM) height profile of the monolayer J‐MoSSe. The high point of the spectrum is attributed to the edge bulge of the sample, which could be a bilayer of J‐MoSSe. To confirm the crystal lattice of the samples, the Raman spectra of the sapphire substrate and two different phases of TMDs, MoS_2_, J‐MoSSe, and A‐MoSSe are recorded as shown in Figure 1e–g. The Raman peak of the sapphire substrate is located at ≈416 cm^−1^. The MoS_2_ sample (Figure 1e) exhibits both out‐of‐plane $A_{1}^{1}$ (at ≈406 cm^−1^) and in‐plane $E^{2}$ (at ≈383 cm^−1^) modes at the peak positions. The J‐MoSSe sample (Figure 1f) exhibits out‐of‐plane $A_{1}^{1}$ (at ≈289 cm^−1^) and in‐plane $E^{2}$ (at ≈354 cm^−1^) and an additional small peak out‐of‐plane $A_{1}^{2}$ (at ≈447 cm^−1^) modes at the peak positions. The A‐MoSSe sample (Figure 1g) retains the original out‐of‐plane $A_{1}^{1}$ and $A_{1}^{2}$ modes and two additional of MoSe_2_‐like out‐of‐plane $A_{1}^{'}$ (at ≈241 cm^−1^) and in‐plane $E^{\prime }$ (at ≈282 cm^−1^) modes at the peak positions, which are consistent with previous literature.^[^ ^54^ ^]^

**Figure 2:**
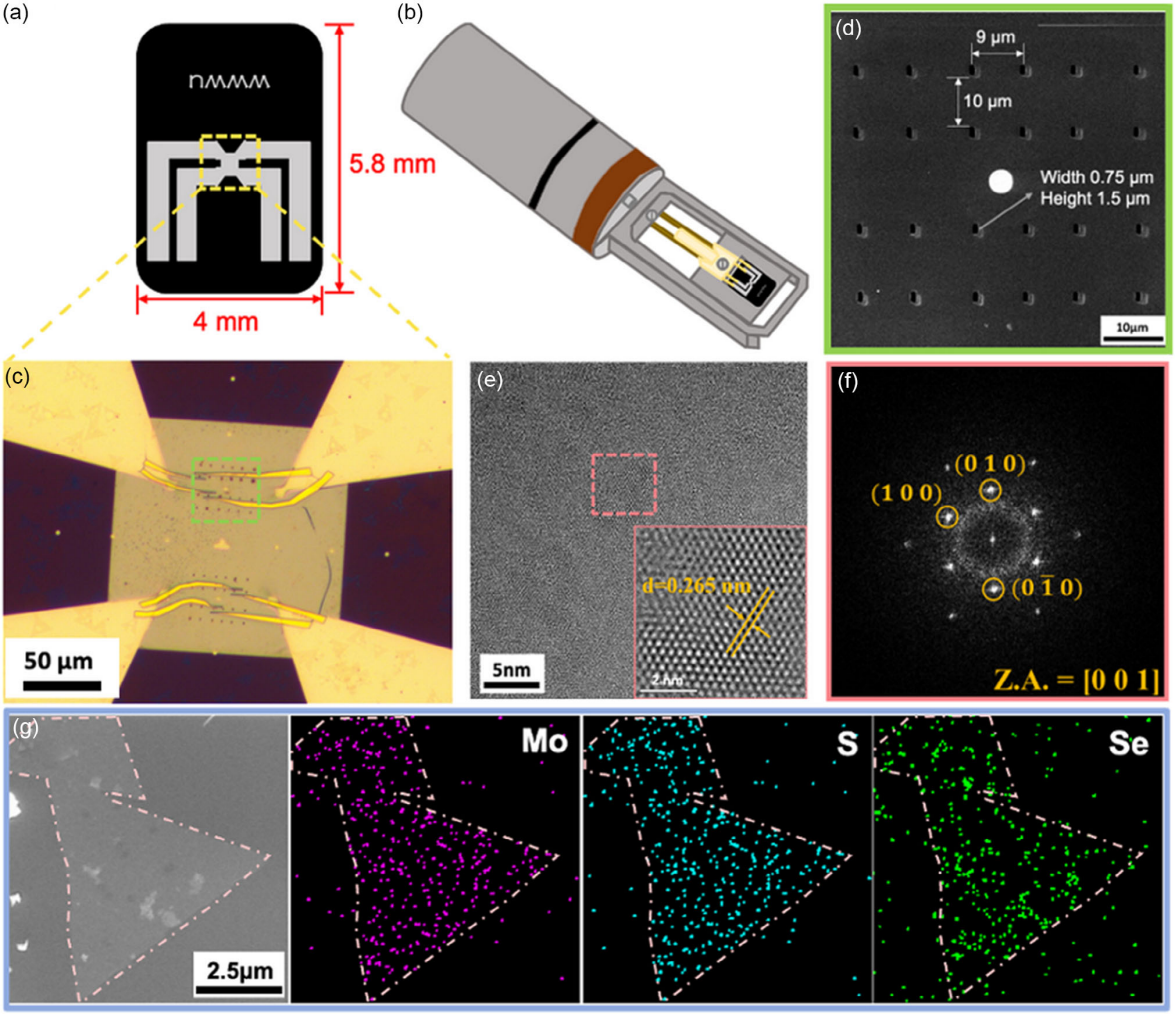
Experimental setup before biasing and basic identification of MoSSe sample. a,b) Schematic of the special electrifying in situ transmission electron microscopy (TEM) chip and holder. c) An OM image of the MoSSe device. d) SEM image of the magnified electrifying TEM chip of the region indicated using green dotted lines in (c), showing an observation window of TEM processed by a focused ion beam with 1.5 μm long and 0.75 μm wide. e) High‐resolution TEM (HRTEM) image of J‐MoSSe and inset shows magnified image for the atomic structure of the pink box. f) Corresponding fast Fourier transform (FFT) along [001] zone axis. g) Scanning transmission electron microscopy (STEM) image and energy‐dispersive X‐Ray spectroscopy (EDX) mapping of the J‐MoSSe sample.

**Figure** **2** shows the experimental setup and the basic identification of the MoSSe sample. Subsequently, the as‐grown MoSSe samples were transferred onto specialized TEM electrical chips (Figure 2a) using a wet transfer process. The electrical chip was then mounted on an in situ TEM holder (Protochips Aduro300), as shown in Figure 2b. The specialized microelectromechanical system (MEMS) chip with a silicon nitride membrane was then processed by a focused ion beam to create an array of holes with 1.5 μm long and 0.75 μm‐wide (Figure 2d). The as‐grown MoSSe was subsequently transferred onto these chips and examined by SEM (Figure S3a, Supporting Information). After SEM inspections, we selected the target area for Au electrode patterning using electron‐beam lithography (Figure S3b, Supporting Information). A plane‐view OM image of the device on specialized TEM electrical chips is shown in Figure 2c, and a cross‐sectional view schematic of the MoSSe device is shown in Figure S1c, Supporting Information. The low‐magnification TEM image of the sample in the observation window was filmed using JOEL F200 TEM (Figure S3c, Supporting Information). The atomic structure of the sample identified using the HRTEM image revealed that the d‐spacing of J‐MoSSe is ≈0.265 nm, with corresponding fast Fourier transform diffraction pattern (FFT‐DP) along [001] zone axis. The contrast of 2D materials is too weak to be recognized by TEM; thus, we used STEM images for identification. Energy‐dispersive X‐Ray spectroscopy (EDX) mapping and elemental atomic percentage reveal that the elements in the samples are homogeneously distributed (Figure 2g and S4, Supporting Information).

**Figure 3:**
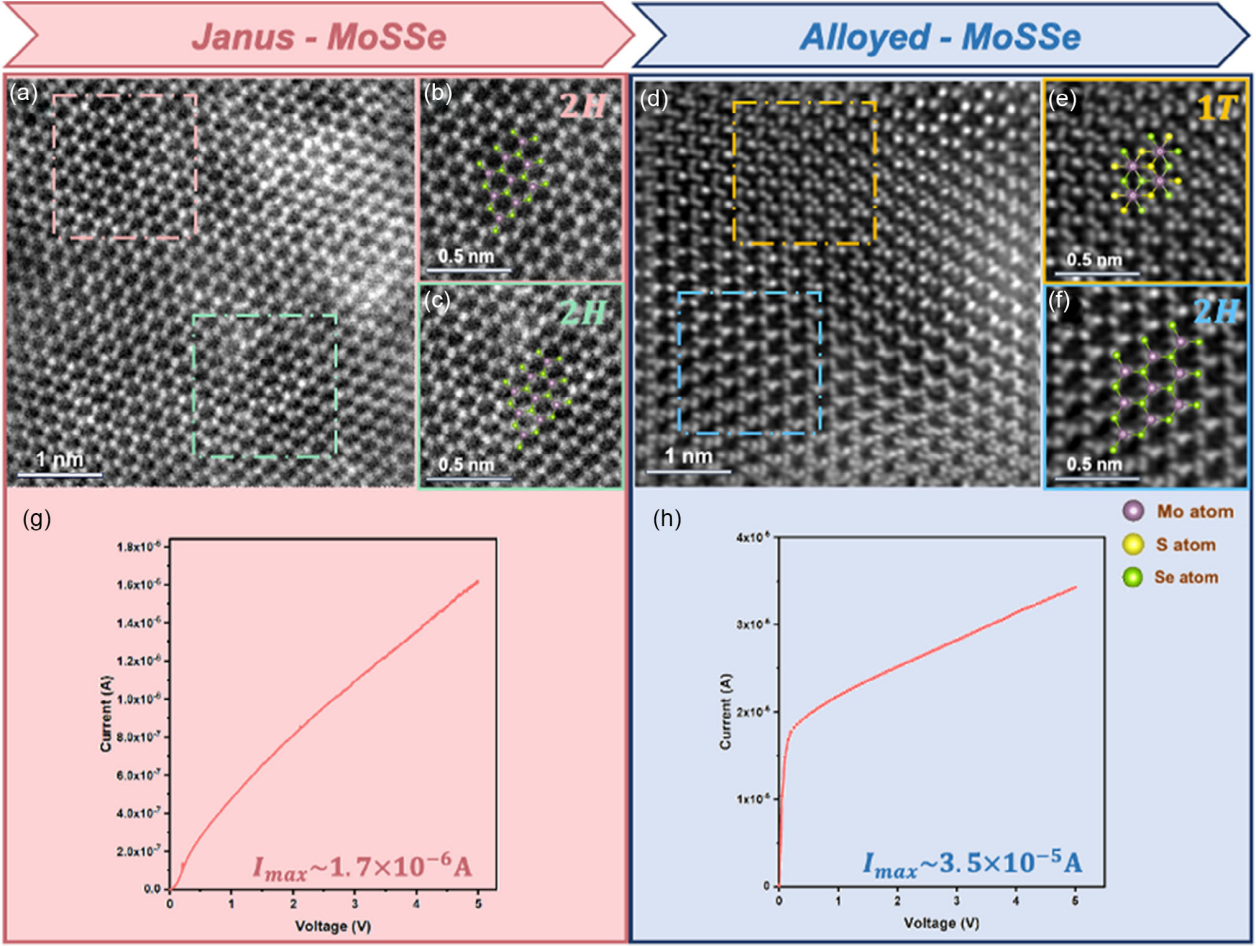
Annular dark‐field STEM (ADF‐STEM) images showing different phases of MoSSe sample and current–voltage (*I–V*) characteristics curve. a) High‐resolution ADF‐STEM images of the J‐MoSSe. b,c) Magnified ADF‐STEM images of the regions indicated in pink, and green in (a), respectively. Displayed 2H hexagonal structure of MoSSe. d) High‐resolution ADF‐STEM images of the A‐MoSSe showing mixed 2H hexagonal and 1T trigonal structures of MoSSe. e) Magnified ADF‐STEM images of the region indicated in yellow in (d), showing 1T trigonal structure of MoSSe. f) Magnified ADF‐STEM images of the region indicated in blue in (d), showing 2H hexagonal structure of MoSSe (as schematically shown in the center where S (yellow), Se (green), and Mo (purple) become displaced). g,h) *I–V* characteristics curves of J‐MoSSe and A‐MoSSe devices under different selenization temperatures of the biasing experiment under 5 V presenting the maximum current value.

At different selenization temperatures, the synthesized MoSSe exhibited a phase transition. According to the high‐resolution ADF‐STEM images, a semiconducting 2H phase was observed in J‐MoSSe (**Figure** **3**a–c). When the selenization temperature increases, the A‐MoSSe phase transitions from the semiconducting 2H phase to the metallic 1T phase. In addition, the semiconducting 2H and metallic 1T phases coexisted in the structure (Figure 3d–f). The electrical performance of J‐MoSSe was similar to that of semiconductor Ohmic contacts (Figure 3g). In contrast, when the 1T metastable phase appeared in A‐MoSSe, the current was significantly increased; the maximum current was an order of magnitude higher than that of J‐MoSSe, and the electrical behavior was similar to that of the metal (Figure 3h). When the selenization temperature is lower (200 °C), the atomic structures are still dominated by the semiconducting 2H phase, i.e., J‐MoSSe (Figure S4a–c, Supporting Information). In terms of the electrical properties, the maximum current decreased with decreasing selenization temperature (Figure S4d, Supporting Information).

The in situ biasing experiment performed at 5 V is first introduced, and all the in situ biasing experiments in this work were conducted with an electron dose of ≈90 pA cm^−2^ and at 800 k time magnification. The applied voltage was increased by 0.05 V s^−1^ compared to the target voltage. Therefore, we unified the electron beam conditions, which also helped us differentiate between the effects of electron beams and biasing. Figure S5, Supporting Information, shows a void etched by electron beam irradiation for 5 min; no crack formation is observed at the void edges.

**Figure 4:**
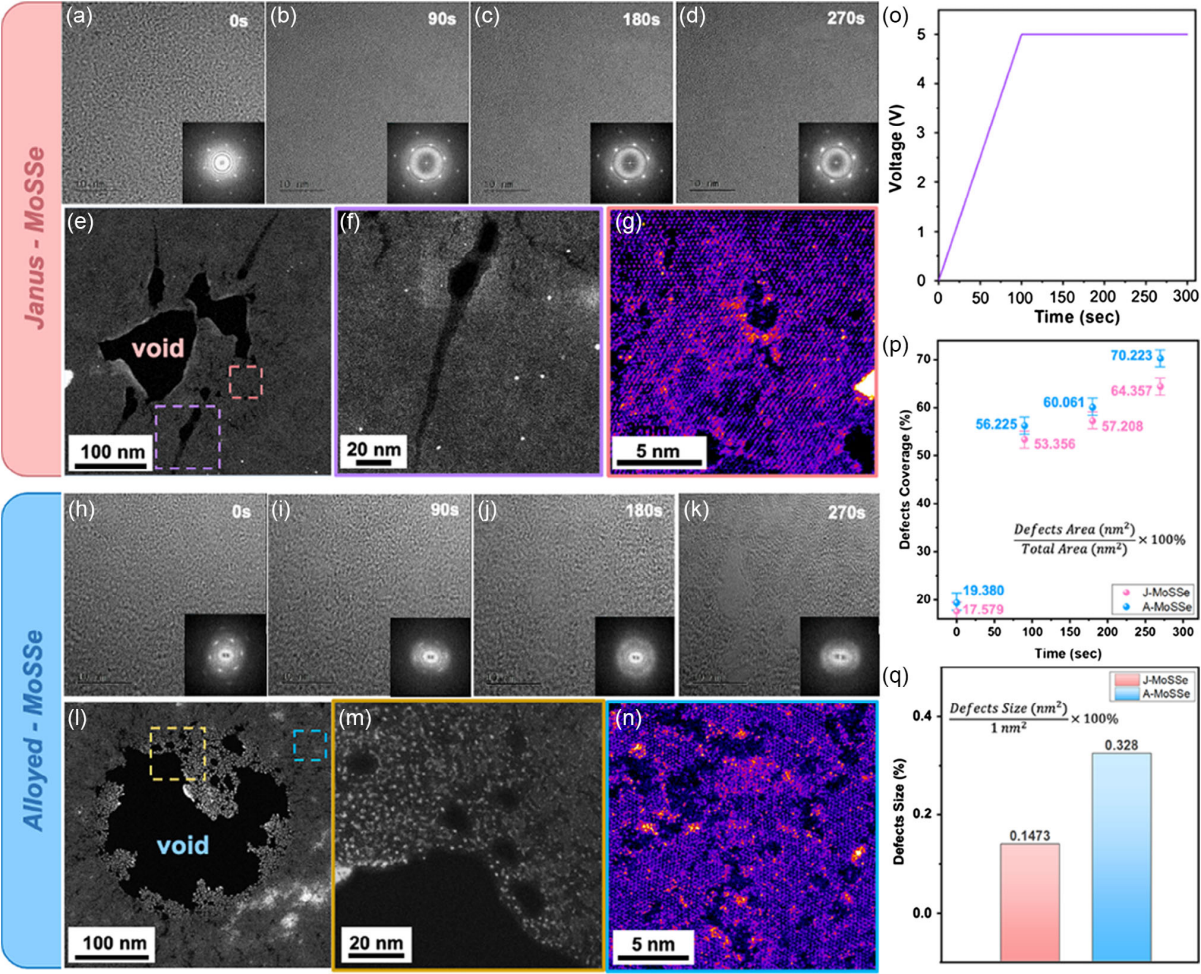
In situ TEM observation of the biasing experiment and ADF‐STEM images showing different phases of damaged MoSSe after 5 V biasing for 5 min. Time‐sequencing TEM images showing significant damage due to the e‐beam and bias. The inset shows the corresponding FFT that reveals the variation in the crystallinity of the sample. a–d) Evolution results for J‐MoSSe. e) Low‐magnification ADF‐STEM images of voids. f) Magnified ADF‐STEM image of the region indicated in purple in (e). g) Pseudo‐colored images of the area outside the e‐beam irradiation scope and magnified images of the region indicated in pink in (e). h–k) Evolution results for A‐MoSSe. l) Low‐magnification ADF‐STEM image of voids. m) Magnified ADF‐STEM images of the region indicated in yellow in (l). n) Pseudo‐colored image of the area outside the e‐beam irradiation scope magnified image of the region indicated in blue in (l). Z‐contrast imaging provides a better understanding of the defect distribution. o) Applied voltage versus time profile of the MoSSe device. p) Point graph with error bars showing the defect coverage percentage of *in situ* observation of the biasing experiment. q) Bar chart showing the defects size percentage in different phases of MoSSe.

**Figure** **4**a–d and h–k shows a series of time‐sequencing TEM images of J‐MoSSe and A‐MoSSe obtained during the in situ experiment, which was performed at 5 V for 5 min, and are shown in Movie S1 and S2, Supporting Information. With different phase structures, the variation in the crystallinity of the samples changed. With the applied bias, the corresponding FFT changed from crystalline to amorphous because of the crystal structure was etched by the electron beam and biasing. For the same biasing time, the J‐MoSSe sample exhibited better crystallinity. In contrast, the A‐MoSSe sample was more easily destroyed and formed voids owing to more current generation. To distinguish the respective contributions of electron beam irradiation and biasing, we also conducted ex situ experiments under the same biasing conditions to minimize the irradiation contribution (Figure S7, Supporting Information). Through the comparison of in situ (with e‐beam) and ex situ (without e‐beam) conditions, it is clear that the crack mainly was promoted by biasing, while the irradiation mainly accelerated the damage of the material.

Detailed analysis of the morphology after biasing was performed using ADF*‐*STEM images. Figure 4e,l shows low‐magnification ADF‐STEM images of typical voids and cracks induced by the electron beam and bias. Furthermore, the sizes of the voids and cracks varied with the phase. We observed that the voids caused by e‐beam irradiation were the largest in A‐MoSSe owing to the increased selenization temperature. Figure 4f,m shows the cracks caused by Joule heating. J‐MoSSe exhibited long and sharp cracks. In addition, when the selenization temperature was lower (200 °C), a series of time‐sequencing TEM images during the in situ experiment are revealed in Figure S8a–d and Movie S3, Supporting Information. The crack morphology was still the same as that of J‐MoSSe, but the crack length was shorter, owing to fewer defects caused by the lower selenization temperature (Figure S8e–g, Supporting Information).

In contrast, the cracks formed in A‐MoSSe were short and truncated. Mo clusters were directly observed at the edge of the void. Because S and Se atoms migrate more easily than Mo atoms, the remaining Mo atoms tend to aggregate to form nanoclusters owing to the differences in the activation barriers for migration between Mo and S atoms. As previous DFT calculations indicate,^[^ ^55^, ^56^ ^]^ the activation barrier for diffusion through intrinsic line defects in MoS_2_ offers a low‐energy pathway for the migration of Mo and S atoms, which are ≈1.6 and 0.7 eV, respectively, and the migration of Mo atoms acts as the rate‐limiting step in this process. The Mo clusters were similar to those observed by Murthy et al. and Chen et al. when MoS_2_ was biased and heated, respectively.^[^ ^53^, ^56^ ^]^ Figure 4g,n shows the defect distribution in the area outside the e‐beam irradiation scope. Figure S6 and S7, Supporting Information, depict additional TEM and ADF‐STEM images of the J‐MoSSe and A‐MoSSe samples after biasing, respectively. We also observed that cracks and defects were spread over several areas of the samples. Figure 4o shows the applied voltage as a function of time. The defect coverage (*D*_c_) of J‐MoSSe and A‐MoSSe is expressed as follows

(1) $$D_{c}=\frac{\text{Defect area }\left(nm^{2}\right)}{\text{Total area }\left(nm^{2}\right)}\times 100\%$$

where the defect area was quantified according to Figure 4a–d and h–k, plotted versus time, and shown in Figure 4p. Simultaneously, the defects coverage of A‐MoSSe increased faster than that of J‐MoSSe because the current was larger and the selenization temperature of the synthesis was higher, resulting in the formation of more defects on the surface. Finally, the defect size was quantified according to Figure 4g,n and as shown in Figure 4q. The size of the defect increased with an increase in the selenization temperature. Adjusting the synthesis temperature of the samples with different phases resulted in biased samples with different morphologies of the voids and cracks.

**Figure 5:**
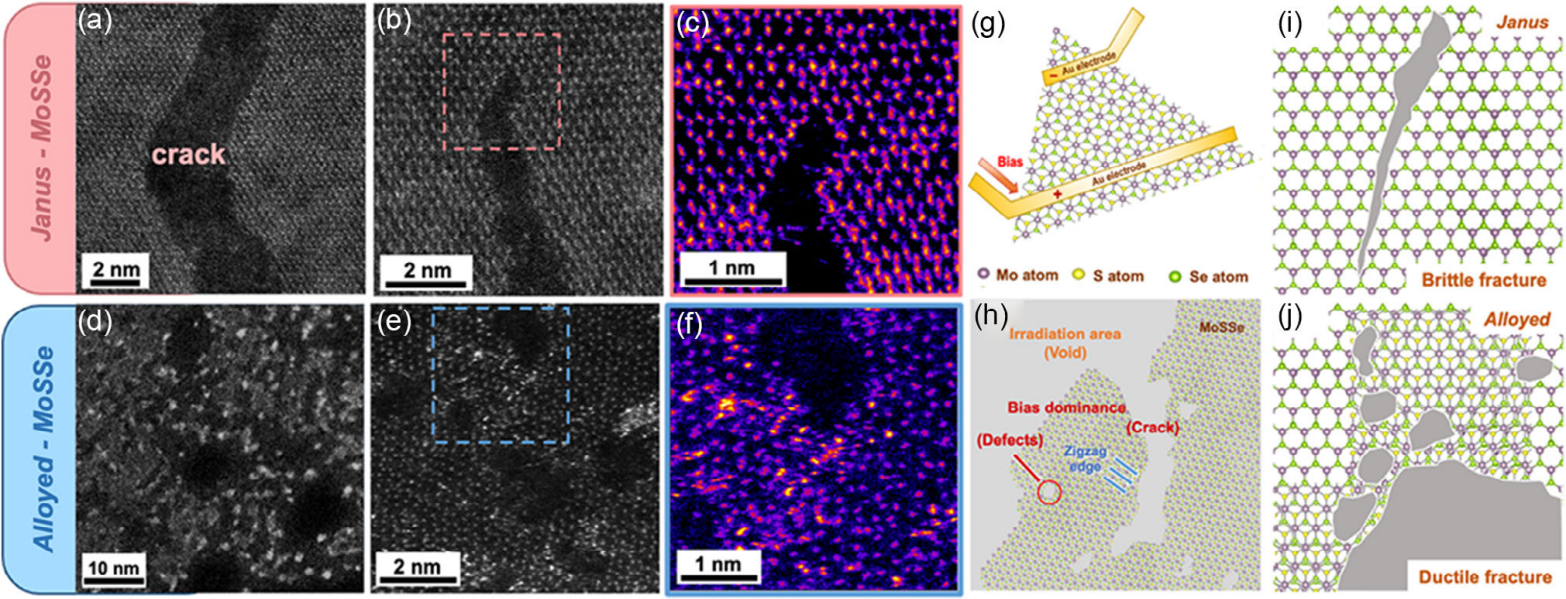
ADF‐STEM images of the bias‐induced cracks and tips of different phases MoSSe and mechanistic study of the dynamic behavior induced by in situ biasing. a,d) ADF‐STEM images of the middle of cracks. b,e) ADF‐STEM images of the tip of cracks. c,f) Magnified ADF‐STEM images of the regions indicated in pink, blue in (b) and (e), respectively. The Z‐contrast imaging provides a better understanding of the arrangement of atoms. g) Schematic of the biasing experiment. h) Schematic of the morphology after biasing showing the formation of voids, cracks, defects. i,j) Schematic of the crack types after biasing. J‐MoSSe is prone to brittle fracture as shown in (i), and A‐MoSSe is prone to ductile fracture as shown in (j).

Furthermore, to better understand the effect of the bias on the device, we intricately analyzed the morphology of the cracks and focused on the middle and tips of the cracks. The J‐MoSSe sample exhibited elongated cracks from the middle (**Figure** **5**a,b). When the crack was formed, the distance between each defect was short; therefore, the probability of encountering a defect was higher, and the crack was prone to migration, which changed its direction. The left and right sides from the boundary of the crack are symmetrical. In addition, from the edges of the fracture cracks, we observed that the atoms had a zigzag arrangement (Figure S6e, Supporting Information). In contrast, the cracks formed in the A‐MoSSe sample were short and point‐like (Figure 5d–f) because the defect concentration was too high to restrict crack propagation after biasing, and significant Mo clusters could be observed at the edge of the voids. Similarly, the tips of the cracks show that the J‐MoSSe sample breaks directly without any remaining atomic bonds (Figure 5c). In addition, residual bonds occurred at the tip of the low‐selenization‐temperature sample (Figure S8h–j, Supporting Information), which was related to a lower defect concentration and a shorter distance between the two defects. The energy provided by the bias is insufficient to allow the atoms to migrate forward.

Finally, a better understanding of the damage of the device at high voltage is required. We also conducted biasing experiment on the J‐MoSSe sample at different voltages (Figure S9, and Movie S4, Supporting Information). At high voltages, folding was observed at the edge of the sample, accompanied by cracks (Figure S10a,b, Supporting Information). In addition, the fracture of the high‐voltage boundaries was smoother and cleaner than that of the low‐voltage boundaries (Figure S11, Supporting Information), and applying a high voltage gave the atoms more energy to migrate. However, the size of the defects also increases, indicating that biasing will increases the defects concentration in the sample (Figure S10c,d, Supporting Information).

Based on the previous discussion of the two etching systems, the dynamic process of the in situ biasing experiment is presented by the manually created atomic model in Figure 5g,h. The reaction is divided into two parts: synthesis and biasing. In the early stage of the reaction, e‐beam irradiation has the capability to displace atoms from their lattice positions, leading to the formation of vacancies and other defects, resulting in many dangling bonds and changes in electrical properties and structural damage. Because atoms with dangling bonds have a lower diffusion barrier, atomic migrations is expected to occur around the defects. Similar to electronic devices operating under normal conditions for long time may generate additional heat and electrical stress, which can also result in the accumulation of point defects, further affect the mechanical, electrical, and thermal properties of the material. After the bias voltage was applied, the current generated by J‐MoSSe was small. Moreover, because of the low temperature during synthesis, the defect concentration is low, which further results in the formation of rapid and long fractures that tend toward brittle fracture. A higher current was generated for the A‐MoSSe sample, allowing the atoms to gain more energy. Subsequently, the S and Se atom depletion and Mo aggregation led to the formation of Mo nanoclusters at the void edges. After the bias dominates, energy is provided for the outward migration of S and Se atoms into line defects, causing some plastic deformation around the tip, which slows down crack propagation and leads to ductile fracture.

## 3 Conclusions

We studied the structural evolution of monolayer polymorphic MoSSe using powerful in situ TEM for biasing experiments. The MoSSe sample was significantly damaged by the bias and showed voids and cracks. We suggest that the Joule heating induced by biasing is the main cause of this evolution. J‐MoSSe and A‐MoSSe prepared at different selenization temperatures exhibited different electrical properties after biasing, which led to diverse mechanical fracture behaviors. A‐MoSSe forms a 1 T metastable state and easily forms dislocations at the two‐phase interfaces, resulting in more surface defects and difficulty in forming long cracks. In contrast, J‐MoSSe exhibits higher crystallinity and forms sharp cracks via brittle fracture. Moreover, with different induced voltage biases, a higher voltage caused more cracks and defects. Applying a high voltage provides the atoms with more energy to migrate. Therefore, the fracture of the high‐voltage boundaries was smoother and cleaner than that of the low‐voltage boundaries. Furthermore, the defects coverage of polymorphic MoSSe was calculated to elucidate the differences in migration ability. By exploring the combination of various synthesis and bias conditions, we provide a comprehensive study on the function of polymorphic MoSSe devices and improve the existing technology.

## 4 Experimental Section

4.1

4.1.1

##### CVD Growth of Monolayer MoS_2_

Sulfur powders (99.5%, Alfa) were placed in the front‐heating zone upstream, and MoO_3_ powders (99%, Aldrich) were positioned in the center‐heating zone of the furnace for CVD. The temperature of the center‐heating zone was gradually increased to 850 °C and maintained for 30 min. Sapphire substrates were placed 2.5 cm far from precursors at the downstream side. During this process, the Ar flow rate was set to 200 sccm. After the reaction, the furnace was allowed to cool down naturally to room temperature.

##### PACVR Process of Monolayer MoSSe

Plasma was held at 35 W for 10 min in PACVR. The Se tank was heated to 200 °C (Janus), 300 °C (Janus), or 450 °C (alloyed). Sapphire substrates were placed in the substrate tank heated to 250 °C and maintained for 40 min. During this process, the H_2_ flow rate was tuned to 250 sccm.

##### Transfer Method of Monolayer MoSSe Samples

A wet transfer method using poly(methyl methacrylate) (PMMA) was employed to transfer the as‐grown MoSSe onto specialized TEM electrical chips. PMMA was spun onto sapphire and subsequently soaked in an NH_4_OH solution (NH_4_OH: deionized [DI] water = 17:100) at 110 °C. The PMMA with MoSSe films was then rinsed several times with DI water, and removed by acetone at 70 °C for 1.5 min.

##### Fabrication of MoSSe Devices

The MoSSe samples were then dripped onto an in situ TEM electrical chip with Si_3_N_4_ membrane windows using a wet transfer process. Methyl methacrylate (bottom layer) and PMMA (top layer) were spin‐coated onto the chip as positive tone photoresist (PR), followed by baking at 180 °C for 1 min by e‐beam lithography. Ti (20 nm) and Au (70 nm) were deposited as electrodes using an e‐gun evaporation system (E‐gun) as electrodes. Subsequently, PR was removed by liftoff using acetone within 24 h. The distance between the two electrodes was 1 μm.

##### Microstructural Characterization of Materials

The samples were characterized by OM and SEM, AFM, and Raman spectroscopy to study their morphology, to determine the sample thickness, and to examine the characteristics of MoS_2_ and MoSSe, respectively (Figure 1). The structural evolution of the MoSSe device was directly observed using field‐emission TEM (JEOL‐F200) at an accelerating voltage of 200 kV, and the elemental distribution was obtained using EDX (Oxford EDS 100 TLE).

##### TEM/STEM with an Electrical‐Biasing Holder and Image Processing

The in situ experiments were performed *via* a special TEM holder (Protochips Aduro300). Bias was applied to the specimen, and the process was observed via TEM (JEOL JEM‐F200) with an accelerating voltage of 200 kV. The *in situ* system also included a power supply (2616 A System Sourcemeter) and a software controller (Fusion 350 V1.0.0). The real‐time TEM and ADF‐STEM images were captured using a Gatan Ultrascan CCD camera with 25 fps at a full 4 × 4 k resolution under an electron dose of ≈90 pA cm^−2^. ADF‐STEM images were obtained by TEM with a Cs corrector (JOEL JEM‐ARM200FTH) at an accelerating voltage of 200 kV. Image J software was used to process the ADF‐STEM images. For these images, Gaussian blur (2 pixels) was applied, and the original grayscale images with black atom contrast were inverted, and then a “fire” false color was used to improve the visual contrast. Atomic models were constructed *via* VESTA software.

## Conflict of Interest

The authors declare no conflict of interest.

## Supporting information

Supplementary Material

## Data Availability

The data that support the findings of this study are available from the corresponding author upon reasonable request.
