# Supplementary material for: Investigation of Atomic‐Scale Mechanical Behavior by Bias‐Induced Degradation in Janus and Alloy Polymorphic Monolayer TMDs via In Situ TEM
Source: Small Sci. 2023 Nov 21;3(12):2300129. doi: 10.1002/smsc.202300129 (PMC11935987; doi:10.1002/smsc.202300129)
Supplement: Supplementary file 1 — Supplementary Material [file SMSC-3-2300129-s001.zip › smsc.202300129-sup-0001-suppdata-S1.pdf]

Supporting Information

Investigation of Atomic-Scale Mechanical Behavior by Biased-Induced Degradation in Janus and Alloy Polymorphic Monolayer TMDs *via In-Situ* TEM

*Hsin-Ya Sung, Chieh-Ting Chen, Yi-Tang Tseng, Yu-Lun Chueh and Wen-Wei Wu**

List of contents

Figure S1 | **Synthesis of as-grown MoSSe.**

Figure S2 | **SEM images of as-grown MoS_2 and MoSSe samples.**

Figure S3 | **SEM, OM, and TEM images of the transferred sample.**

Figure S4 | **EDX elemental mapping showing the distribution of Mo, S and Se.**

Figure S5 | **ADF- STEM images showing MoSSe sample under low selenization temperature and I–V characteristics curve.**

Figure S6 | **TEM and STEM images of the results of the damage using only electron beam irradiation for 5 min without biasing.**

Figure S7 | **Results of MoSSe under 300 ºC selenization temperature *in-situ* biasing at 5 V.**

Figure S8 | **Results of MoSSe under 450 ºC selenization temperature *in-situ* biasing at 5V.**

Figure S9 | ***Ex-situ* TEM observation of the biasing experiment showing damaged Alloyed-MoSSe after 5V biasing for 5 min.**

Figure S10 | ***In-situ* TEM observation of the biasing experiment and ADF-STEM images of low-selenization-temperature-MoSSe showing damaged MoSSe after 5 V biasing for 5 min.**

Figure S11 | ***In-situ* TEM observation of the biasing experiment under 10 V.**

Figure S12 | **Results of Janus-MoSSe after *in-situ* biasing at 10 V and defect area percentage at different voltages.**

Figure S13 | **Results of bias-induced cracks and tips of Janus-MoSSe at different bias voltages.**

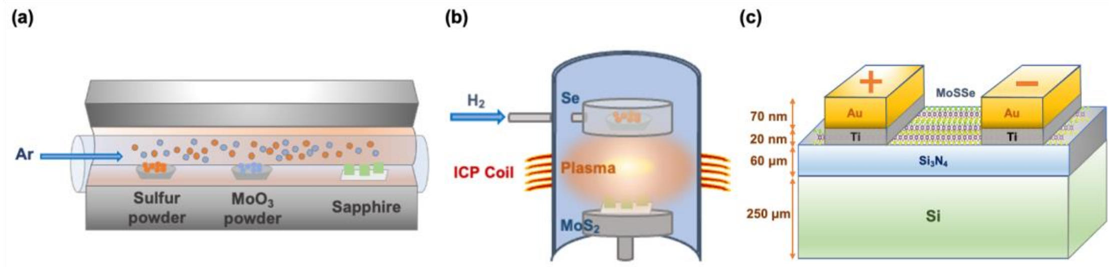

Figure S1. Synthesis of as-grown MoSSe.

(a) Schematic of the experimental set-up for CVD-growth of MoS_2. (b) Schematic of PACVR process. (c) Schematic of the MoSSe device.

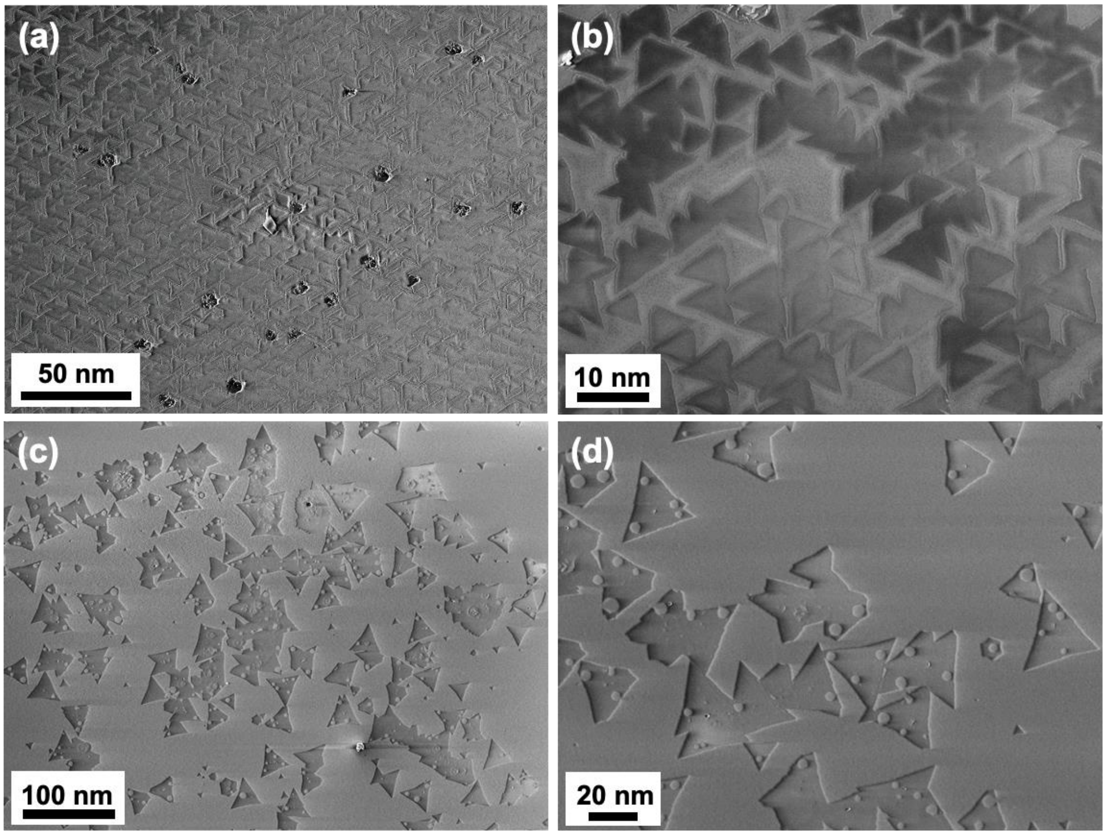

Figure S2. SEM images of as-grown MoS_2 and Janus-MoSSe samples.

(a, b) SEM image of as-grown MoS_2 sample. (c, d) SEM image of as-grown Janus-MoSSe sample.

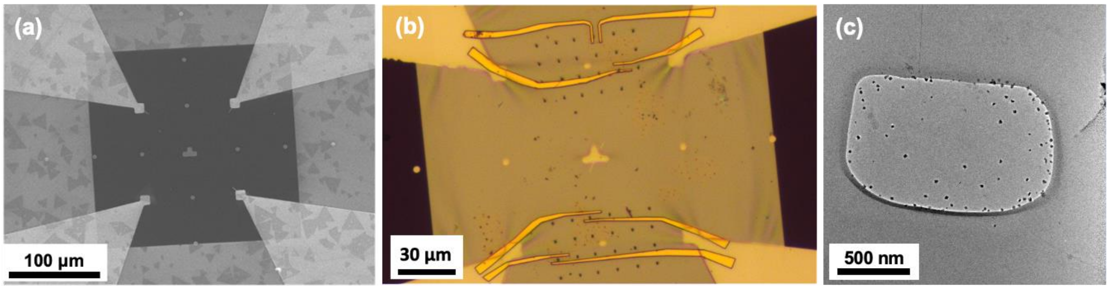

Figure S3. SEM, OM, and TEM images of the transferred sample.

(a) SEM image of as-grown MoSSe sample transferred on specialized TEM electrical chips. (b) OM image of transferred MoSSe sample after electrode fabrication by electron beam lithography. (c) Low-magnification TEM image of MoSSe sample in observation window.

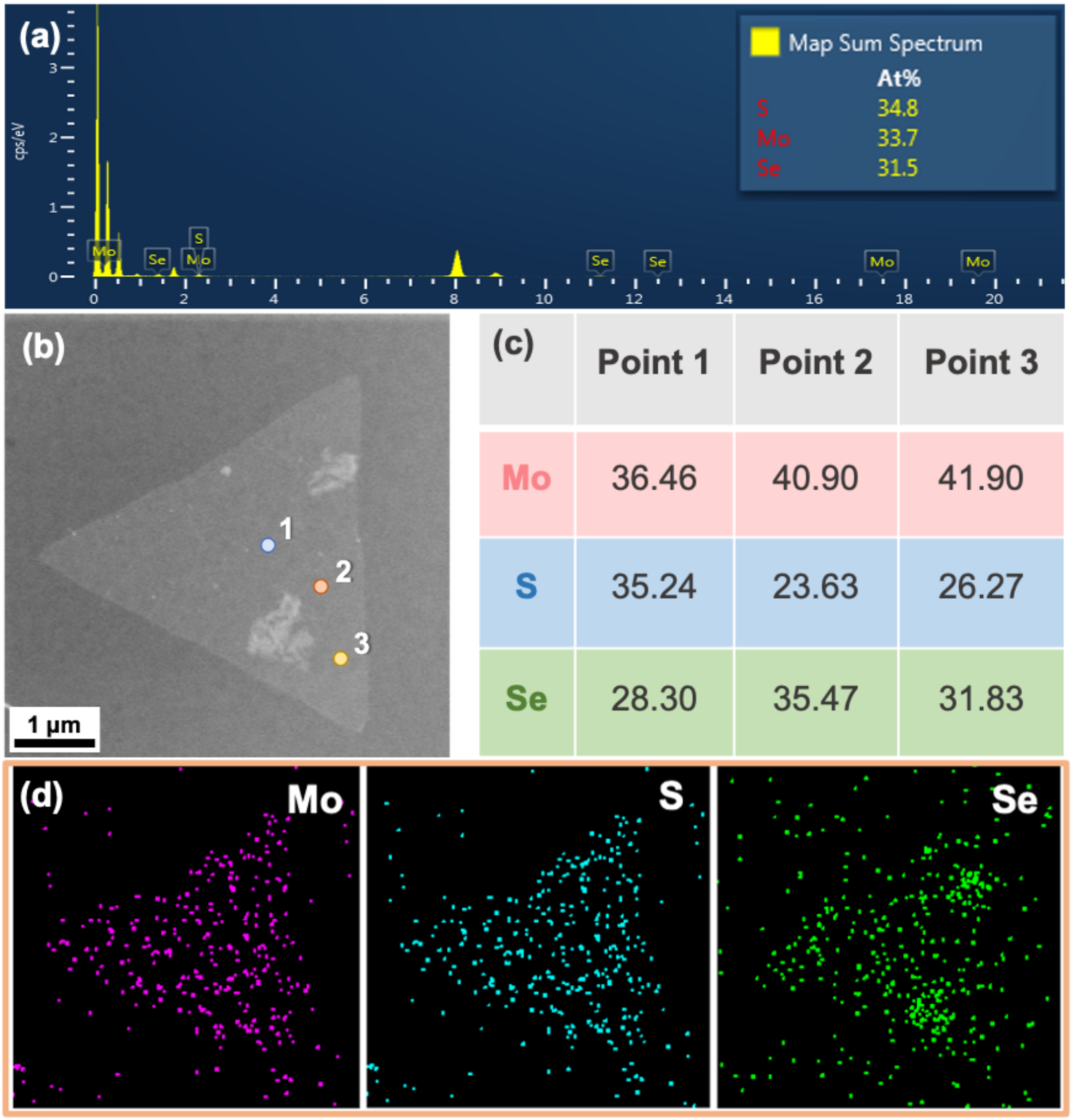

Figure S4. EDX elemental mapping showing the distribution of Mo, S and Se.

(a) Mo, S and Se elemental atomic percentage of the plots shown in Figure 2(g). (b) Low-magnification STEM image. (c) Mo, S and Se elemental atomic percentage of the plots shown in (b). (d) EDX spectrum of the Janus-MoSSe sample.

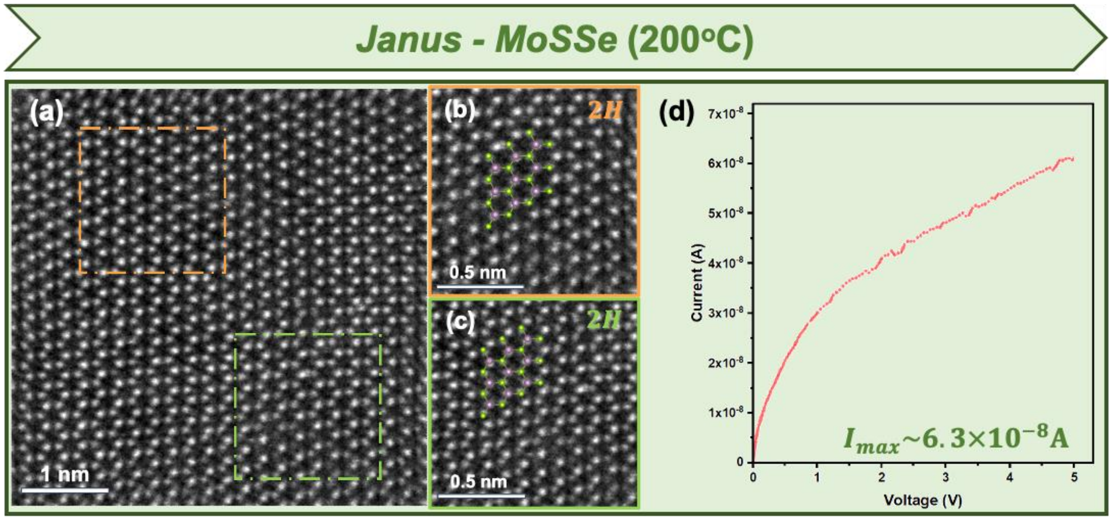

Figure S5. ADF- STEM images showing MoSSe sample under low selenization temperature and I–V characteristics curve.

(a) High-resolution ADF-STEM images of the Janus-MoSSe. (b, and c) Magnified ADF-STEM images of the areas indicated in orange, and green dotted lines in (a), respectively. Displayed 2H hexagonal structure of MoSSe. (as schematically shown in the center where S (yellow), Se (green) and Mo (purple) become displaced). (d) I–V characteristics curve of MoSSe device under selenization temperatures of 200°C the biasing experiment under 5 V and presenting the maximum current.

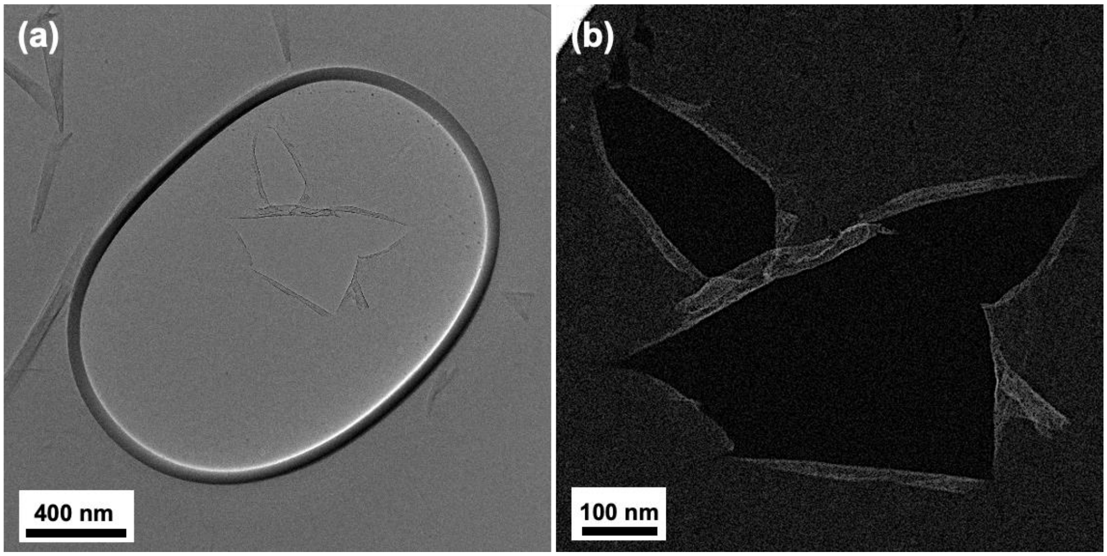

Figure S6. TEM and STEM images of the results of the damage using only electron beam irradiation for 5 min without biasing.

(a) HRTEM image showing the e-beam-induced void in the irradiated area. (b) Magnified ADF-STEM images of the void damaged by electron beam irradiation.

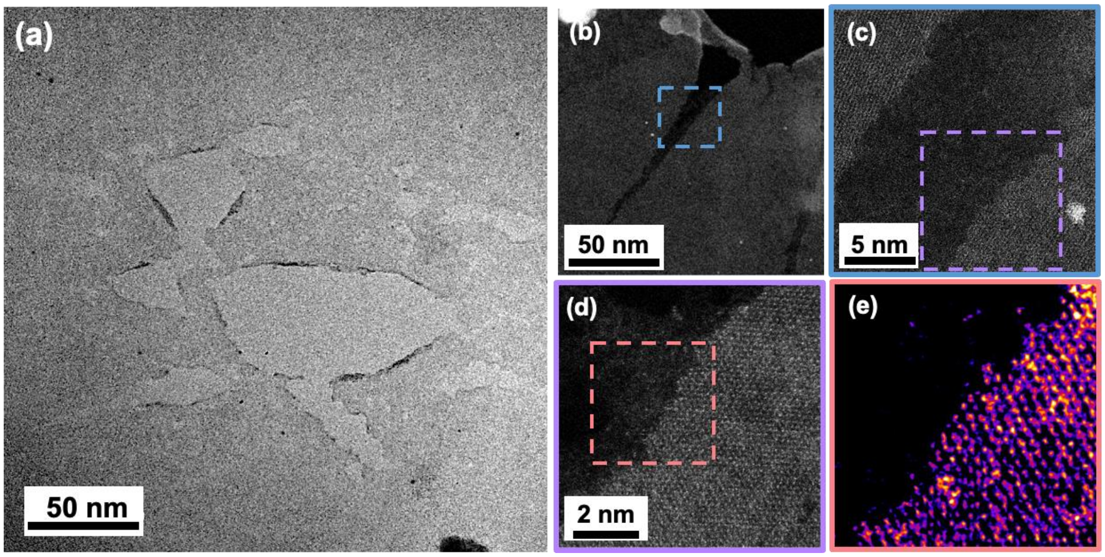

Figure S7. Results of MoSSe under 300 ºC selenization temperature *in-situ* biasing at 5 V.

(a) Low-magnification TEM image of voids caused by electron beam irradiation. (b) ADF-STEM image of cracks. (c) Magnified ADF-STEM image of the region indicated in blue in (b). (d) Magnified ADF-STEM image of the region indicated in purple in (c). (e) Magnified ADF-STEM image of the region indicated in pink in (e), which shows that the observed pseudo-color image of the edge of the crack is in zigzag arrangement.

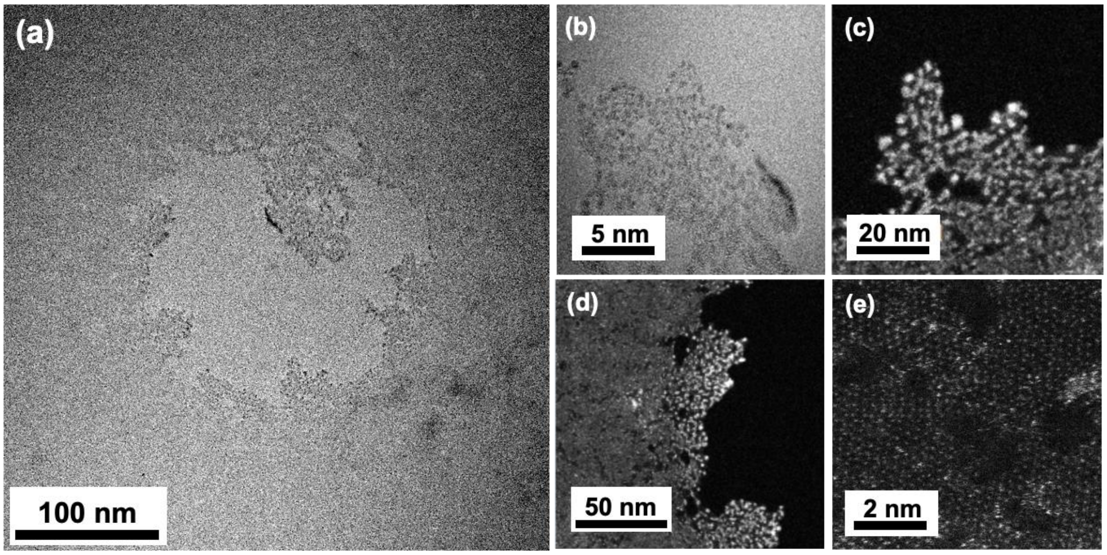

Figure S8. Results of MoSSe under 450ºC selenization temperature *in-situ* biasing at 5V.

(a) Low-magnification TEM image of voids caused by electron beam irradiation. (b) TEM image of the edge of the void decorated with nanoclusters. (c) ADF-STEM image of nanoclusters. (d) ADF-STEM image of the edge of the void. (e) ADF-STEM image of the area outside the e-beam irradiation scope.

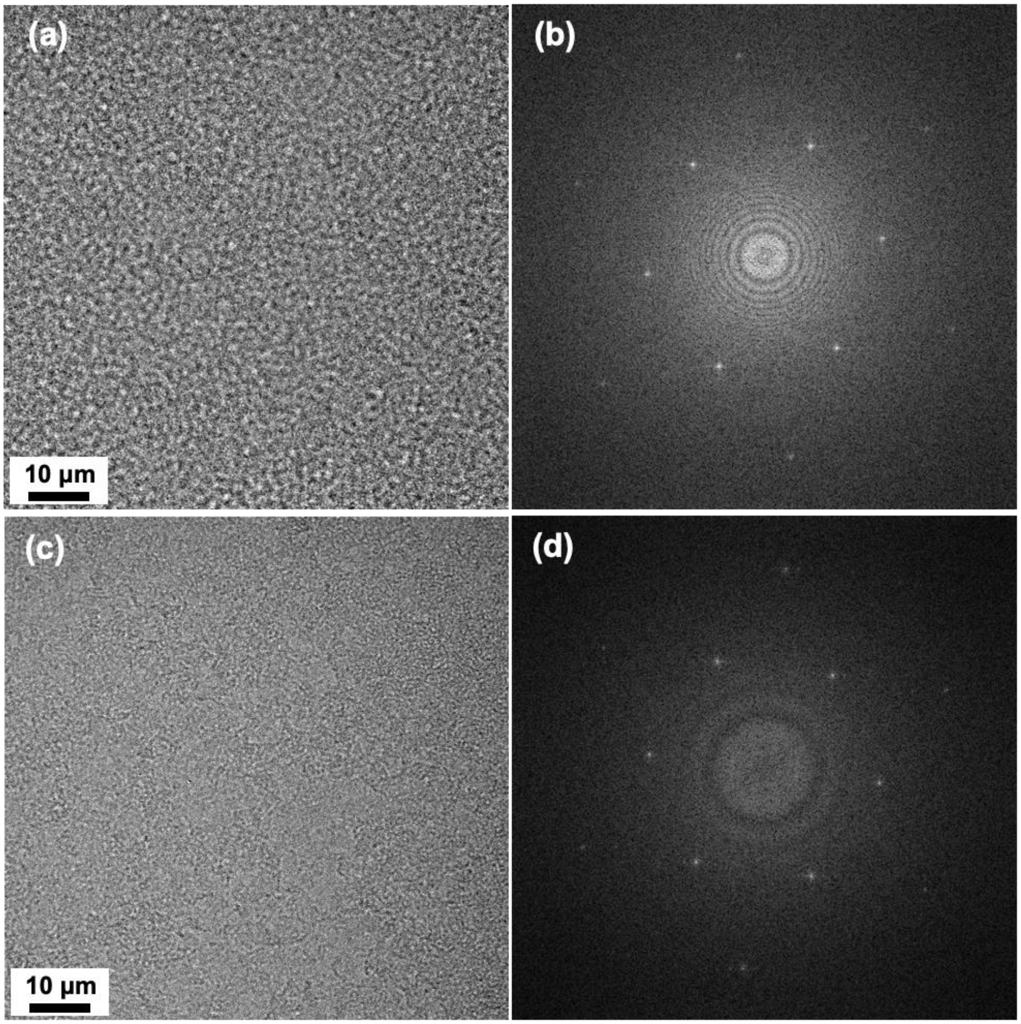

Figure S9. ***Ex-situ*** **TEM observation of the biasing experiment showing damaged Alloyed-MoSSe after 5V biasing for 5 min.**

(a-b) showing the pristine HRTEM and corresponding FFT-DP of A-MoSSe. (c-d) showing the HRTEM and corresponding FFT-DP of A-MoSSe after biasing 5V for 5 min. The applied voltage was increased by 0.05 V per second. In *ex-situ* conditions, only reduce the degree of crystallization of A-MoSSe.

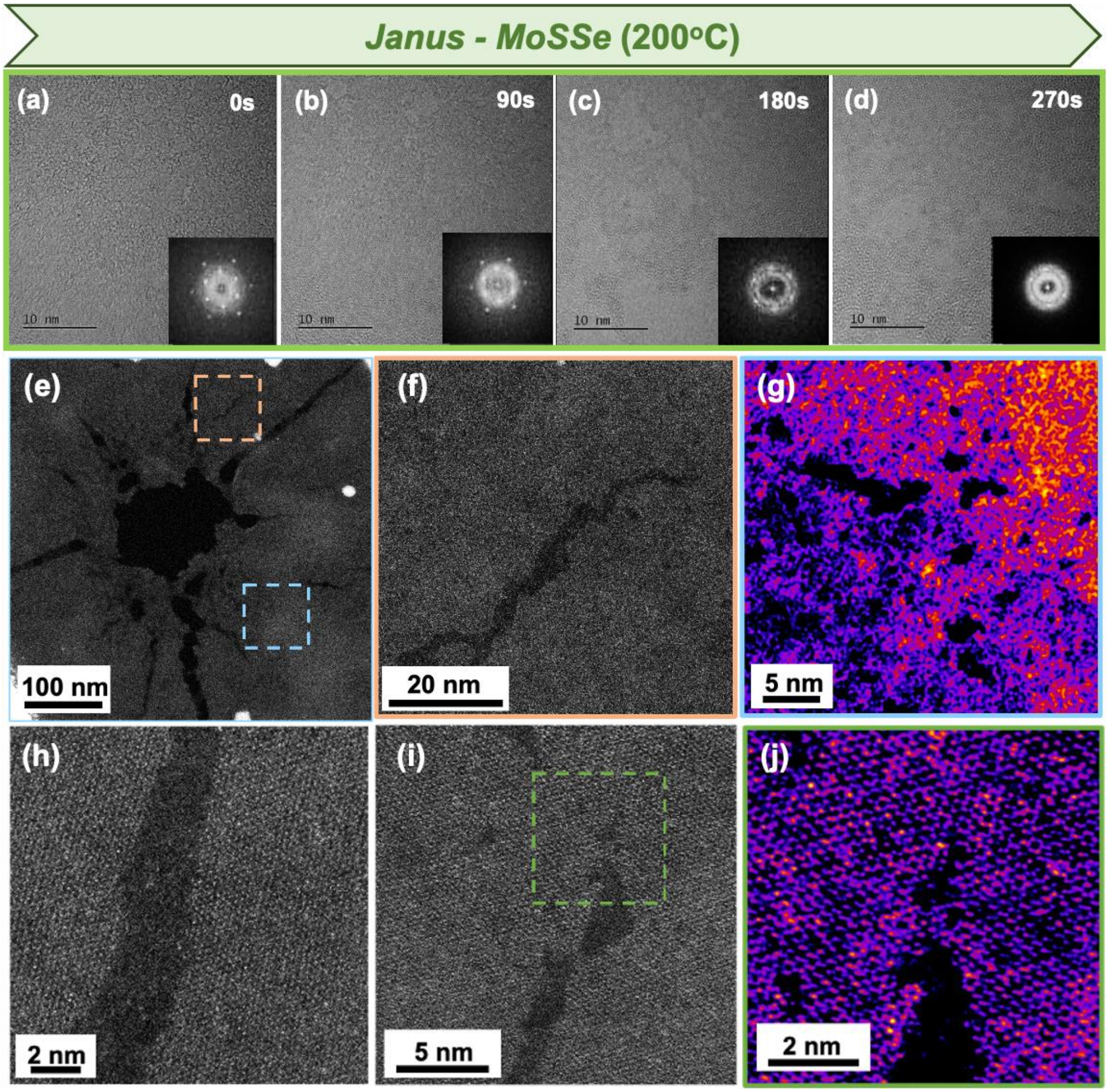

Figure S10. ***In-situ*** **TEM observation of the biasing experiment and ADF-STEM images of low-selenization-temperature-MoSSe showing damaged MoSSe after 5 V biasing for 5 min.**

Time-sequencing TEM images show significant damage to the e-beam and bias. The inset shows the corresponding FFT-DP that reveals the variation in the crystallinity of the sample. (a−d) Evolution results of MoSSe under 200 ºC selenization temperatures. (e) Low-magnification ADF-STEM images of voids. (f) Magnified ADF-STEM image of the region indicated in orange in (e). (g) Pseudo-color images of the area outside the e-beam irradiation scope and magnified images of the region indicated pink in (e). (h) ADF-STEM images of the middle of cracks. (i) ADF-STEM images of crack tip. (j) Magnified ADF-STEM images of the region indicated in green in (i). Pseudo-color image of crack tip. Z-contrast imaging provides a better understanding of the arrangement of atoms.

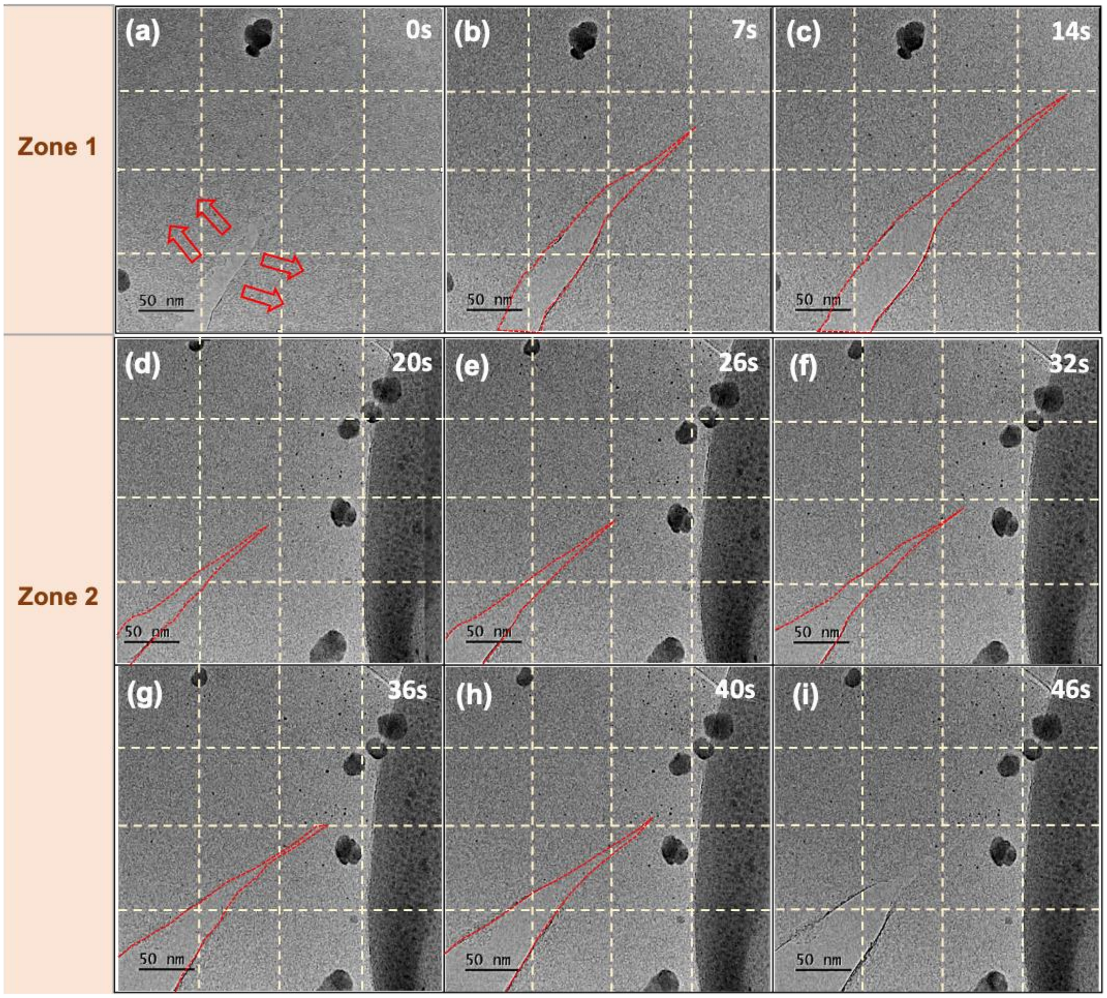

Figure S11. ***In-situ*** **TEM observation of the biasing experiment under 10 V.**

Time-sequencing TEM images showing the migration of the crack tip at two different observation zone. The cracked areas are circled in red for better growth identification. (a) red arrow indicated the direction of the crack migration.

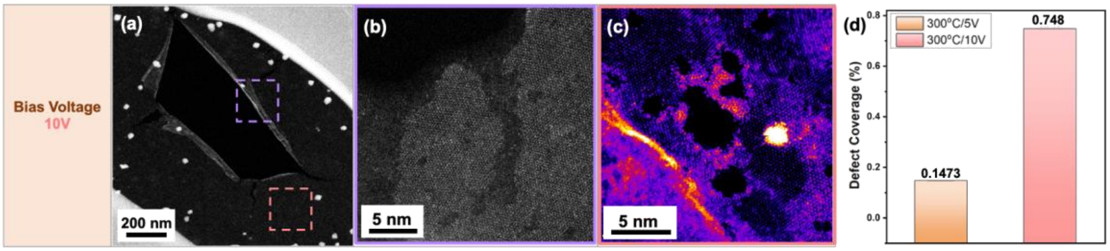

Figure S12. Results of Janus-MoSSe after *in-situ* biasing at 10 V and defect area percentage at different voltages.

(a) Low-magnification ADF-STEM image of voids. (b) Magnified ADF-STEM image of the region indicated in purple in (a). (c) Magnified ADF-STEM image of the region indicated in pink in (a). Pseudo-color image of the area outside the e-beam irradiation scope. Z-contrast imaging provides a better understanding of the distribution of defects. (d) Bar chart showing the defect size percentage at different bias voltages.

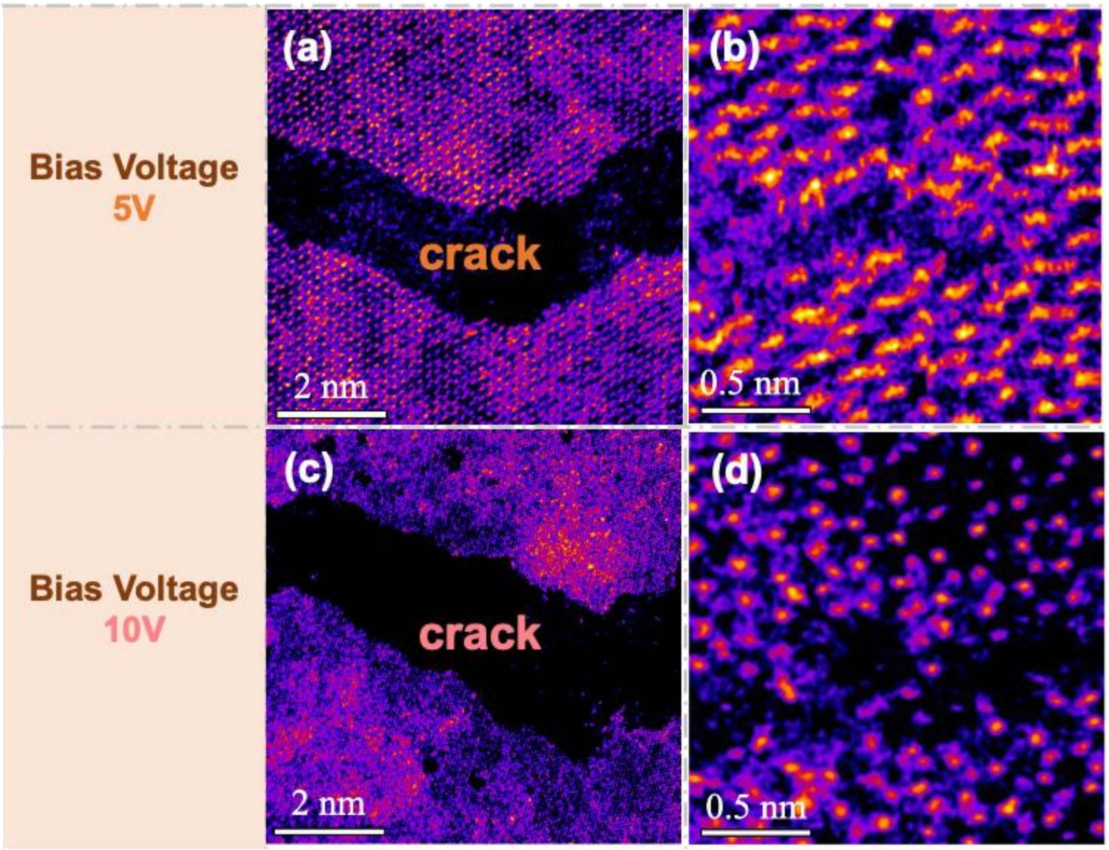

Figure S13. Results of bias-induced cracks and tips of Janus-MoSSe at different bias voltages.

(a, and c) ADF-STEM images of the middle of cracks. (b, and d) ADF-STEM images of the tip of cracks. Z-contrast imaging provides a better understanding of the arrangement of atoms.
